# Treatment of femoral defects in rats with collagen and elastin polymers combined with hydroxyapatite and bone morphogenetic protein

**DOI:** 10.3389/fbioe.2026.1803995

**Published:** 2026-04-08

**Authors:** Eduardo Gomes Machado, Marina Ribeiro Paulini, Gustavo Andrade Fraga, Ana Maria de Guzzi Plepis, Virgínia da Conceição Amaro Martins, Ewerton Alexandre Galdeano, Renato de Moraes, Marcelo de Azevedo e Souza Munhoz, João Paulo Mardegan Issa, Rogério Leone Buchaim, Daniela Vieira Buchaim, Victor Augusto Ramos Fernandes, Geovane Ribeiro dos Santos, Marcelo Rodrigues da Cunha

**Affiliations:** 1 Interunits Graduate Program in Bioengineering (EESC/FMRP/IQSC), University of São Paulo (USP), SãoCarlos, Brazil; 2 Medical Clinic, Faculty of Medicine of Jundiaí (FMJ), Jundiaí, Brazil; 3 Department of Basic and Oral Biology, School of Dentistry of Ribeirão Preto, University of São Paulo (FORP-USP), RibeirãoPreto, Brazil; 4 University of São Paulo (USP), São Carlos Institute of Chemistry (IQSC), SãoCarlos, Brazil; 5 Postgraduate Program in Health Sciences, Faculty of Medicine of Jundiaí (FMJ), Jundiaí, Brazil; 6 Department of Biological Sciences, Bauru School of Dentistry (FOB), University of Sao Paulo (USP), Bauru, Brazil; 7 Graduate Program in Anatomy of Domestic and Wild Animals, Faculty of Veterinary Medicine and Animal Science (FMVZ), University of Sao Paulo (USP), Sao Paulo, Brazil; 8 Medical School, University Center of Adamantina (FAI), Adamantina, Brazil

**Keywords:** biomaterials, bone defects, bone morphogenetic proteins (BMPs), hydroxyapatite (HA), tissue engineering

## Abstract

Biomaterials have emerged as promising therapeutic alternatives in clinical conditions in which bone regenerative capacity is limited, whether due to trauma or pathological bone loss. Among these, collagen-based materials, hydroxyapatite (HA), and bone morphogenetic proteins (BMPs) have been extensively investigated, while elastin has more recently gained attention as a substrate for tissue regeneration. This study aimed to evaluate bone repair in femoral defects in rats treated with elastin- and collagen-based membranes subjected to controlled alkaline hydrolysis under different processing conditions, used alone or combined with HA or recombinant human BMP-2 (1.1 µg per defect). A total of 77 rats underwent surgical creation of a non-critical unicortical femoral defect (3 mm diameter) and were allocated into seven groups: defect without graft (control); elastin membrane; elastin membrane + HA; elastin membrane + BMP-2; porcine intestinal serosa–derived collagen membrane; collagen membrane + HA; and collagen membrane + BMP-2. Histological analyses confirmed the biocompatibility of all membranes, with no evidence of inflammatory response. Elastin membranes, when used alone or combined with HA, did not significantly enhance bone repair compared with the control group; however, their association with BMP-2 improved osteogenesis. In contrast, collagen membranes, whether used alone or combined with HA or BMP-2, demonstrated superior bone formation and integration. In conclusion, the evaluated biomaterials exhibited osteogenic potential in non-critical femoral defects, and BMP-2 significantly enhanced outcomes, particularly when combined with collagen-based scaffolds.

## 1 Introduction

Osteogenesis, or new bone formation, is a complex process that occurs in bone defects and is influenced by several factors, including time, physiological conditions, and the type of defect (Suh et al., 2023). Although bone tissue exhibits plasticity and regenerative capacity, bone defects often require therapeutic interventions to stimulate the regenerative process (Trompet et al., 2024), particularly in cases of bone mass loss resulting from trauma or clinical conditions such as tumor resection and pathological fractures associated with osteoporosis (Graue et al., 2025). The primary challenge in critical-sized defects is to provide an appropriate and stable platform for bone regeneration that enables cell migration and the formation of new bone tissue (Oh et al., 2023).

Autologous bone grafting, which is taken from the patient’s own body, is considered the “gold standard” in the treatment of bone defects because it contains osteogenic cells and osteoinductive properties (Schmidt, 2021). However, its use has limitations, such as morbidity at the collection site and additional surgical time. Allografts and xenografts emerge as alternatives, but they do not contain living cells and present risks of immune rejection and disease transmission (Dimitriou et al., 2011). Therefore, tissue engineering has emerged as a promising approach for the development of bone substitutes, using biomaterials and cells to promote bone regeneration (Battafarano et al., 2021).

Biological materials, such as demineralized bone matrix (DBM), are used to induce osteogenesis because they contain bone morphogenetic proteins (BMPs) that activate mesenchymal cells to differentiate into osteoblasts (Ren et al., 2024). The combination of BMPs with suitable biomaterials can enhance bone formation in experimental models. However, controlled release of BMPs is a challenge because their *in vivo* half-life is very short (Kanjilal et al., 2019). Therefore, the use of carriers for BMPs, such as calcium phosphate ceramics, can improve the effectiveness of the treatment (Stokovic et al., 2020).

Hydroxyapatite (HA), a biocompatible material with a structure similar to bone, is often used as a biomaterial in bone defects due to its high biocompatibility and ability to promote neovascularization (Kavitha Sri et al., 2023). HA provides support for cellular migration and the formation of new bone, making it one of the most effective alternatives for treating bone defects. Furthermore, the addition of natural polymers like collagen and elastin has shown promising results in bone regeneration, as these materials mimic the extracellular matrix of bone, facilitating the migration and differentiation of osteogenic cells (Farazin and e Mahjoubi, 2024; Shang et al., 2022).

Elastin was selected as a biomaterial due to its unique structural and mechanical properties that distinguish it from other naturally derived biopolymers. As a key component of the extracellular matrix, elastin provides elasticity and resilience, allowing scaffolds to better accommodate the mechanical stresses present at bone regeneration sites. The main component of elastin is tropoelastin, which mediates essential cellular processes such as cytoskeletal organization, chemotaxis, and proliferation. In addition, elastin-derived matrices support cell adhesion, migration, and osteogenic differentiation, while maintaining low immunogenicity and high biological stability. Elastin also possesses characteristics that allow the incorporation of target cells and signaling molecules involved in extracellular matrix remodeling. Unlike highly hydrophilic polymers such as hyaluronic acid, elastin contributes to the mechanical integrity of the scaffold, which is critical for maintaining defect space and supporting tissue ingrowth during bone healing (P et al., 2022; Coletta et al., 2017).

Collagen, in particular, has stood out as an excellent biomaterial due to its biocompatibility and its ability to act as a carrier for bone-inducing proteins, such as BMPs (Xing et al., 2021; Ren et al., 2022). Studies suggest that the use of collagen combined with hydroxyapatite and BMPs can accelerate bone regeneration, reducing the amount of proteins required for the osteoinduction process. Moreover, chemical treatment of collagen can improve its mechanical and physiological properties, favoring cell adhesion and osteogenesis (Stuckensen et al., 2019).

The combination of materials such as collagen, elastin, and hydroxyapatite in support matrices has proven to be an effective approach for bone regeneration. These biomaterials possess osteoinductive and osteoconductive properties, promoting cellular migration and the formation of new blood vessels, which are essential for the bone healing process (Rodríguez-Merchán, 2022). The mineralization of these biopolymers, combined with calcium phosphate, may be an effective strategy for healing bone defects, offering a promising alternative for the treatment of critical bone injuries (Deshpande et al., 2021).

This study aimed to evaluate the osteoregenerative performance and biocompatibility of elastin- and collagen-based matrices, alone or combined with hydroxyapatite and bone morphogenetic protein (BMP), in a rat femoral bone defect model using histological, morphometric, and biomechanical analyses.

## 2 Materials and methods

### 2.1 Preparation of biomaterials

#### 2.1.1 Elastin matrix

Bovine auricular cartilage was washed with 0.9% saline solution (NaCl) and distilled water, followed by treatment with an alkaline hydrolysis solution containing salts (sulfates and chlorides) and hydroxides of alkali and alkaline earth metals for 24 h at 37 °C. This procedure was carried out by the Biochemistry and Biomaterials Group at the Institute of Chemistry of São Carlos–University of São Paulo (USP), adapted from the method used for collagen extraction (Pal et al., 2023).

Subsequently, the cartilage was equilibrated in a solution containing sulfates and chlorides of Na^+^, K^+^, and Ca^2+^ ions. Excess salts were removed by sequential washing with 3% boric acid solution, deionized water, 0.3% EDTA solution, and again with deionized water. The resulting elastin membrane was equilibrated in phosphate-buffered saline (PBS, pH 7.4) for 24 h, thoroughly washed with deionized water, then frozen and lyophilized. The elastin matrix was cut into 3 mm diameter samples and designated ME.

## 2.1.2 Collagen matrix

The collagen matrix was obtained from porcine intestinal serosa using the same protocol employed for elastin matrix preparation. However, due to the distinct structural characteristics of the materials, the hydrolysis step was performed at different temperatures. Previous exploratory data demonstrated that hydrolysis of porcine intestinal serosa at 37 °C leads to collagen denaturation, whereas this effect does not occur when the process is conducted at 25 °C. Therefore, to preserve the triple-helical structure and biological functionality of collagen, hydrolysis was carried out at 25 °C. In contrast, for elastin obtained from bovine auricular cartilage, processing at 25 °C was insufficient to promote hydrolysis, as this tissue exhibits greater thermal resistance. Consequently, a temperature of 37 °C was selected for elastin processing, enabling effective hydrolysis without material denaturation. The resulting collagen matrix was designated MS.

### 2.1.3 Hydroxyapatite synthesis

Hydroxyapatite (HA) was synthesized using a solution of calcium nitrate [Ca(NO_3_)_2_·4H_2_O], adjusted to pH 11.0 with concentrated ammonium hydroxide, and a solution of ammonium phosphate [(NH_4_)_2_HPO_4_]. The calcium solution was stirred vigorously and maintained under a constant nitrogen flow. The phosphate solution was then slowly added, resulting in the formation of a gelatinous precipitate, which was stirred for 24 h at room temperature (Mishchenko et al., 2023; Horn et al., 2009; Jarcho et al., 1976).

After this period, the suspension was decanted and the supernatant discarded. The resulting HA precipitate was washed with deionized water until neutral pH, centrifuged at 4,000 rpm for 15 min at 20 °C, and dried at 90 °C for 24 h. The dried material was then ground and sieved to obtain particles smaller than 0.149 mm.

Both the elastin and collagen matrices, as well as the hydroxyapatite, were packaged in appropriate containers and sterilized using ethylene oxide by the company Acecil Central de Esterilização Com. Ind. Ltda (Campinas, SP, Brazil).

#### 2.1.4 Characterization

Differential Scanning Calorimetry (DSC): The denaturation temperature (Td) of the matrices was determined from the inflection point of the DSC curves, using a TA Instruments model DSC-2010 device, calibrated with an indium standard. Approximately 15 mg of each sample was sealed in hermetic aluminum pans and analyzed under a nitrogen atmosphere (80 mL min^-1^), with a heating rate of 10 °C·min^-1^, within a temperature range of 5 °C–120 °C.

Scanning Electron Microscopy (SEM): Surface morphology was examined using SEM. The samples were mounted on aluminum stubs with conductive carbon tape and coated with a 6 nm gold layer using a BAL-TEC MED 020 sputter coater (BAL-TEC, Liechtenstein), operated at 2.00 × 10^−2^ mbar, 60 mA current, and a deposition rate of 0.60 nm s^-1^. Micrographs were obtained using a ZEISS LEO 440 microscope (Cambridge, United Kingdom), equipped with an OXFORD model 7,060 detector, operated at an accelerating voltage of 20 kV.

### 2.1.5 Animals and experimental groups

A total of 77 male rats (*Rattus norvegicus*, Wistar), 16 weeks old and with an average weight of 330 g, were used. They were provided by the Biotério da Granja R.G. (Suzano, SP) and IPEN (Institute of Energy and Nuclear Research) and were kept in the animal facility of the Faculty of Medicine of Jundiaí, Jundiaí-SP, Brazil. Four animals were housed per cage and received a balanced diet (Purina-Nestlé Brasil Ltda) and water *ad libitum*. The environment had a controlled temperature (23 °C ± 1 °C) and a 12-h light/dark photoperiod, with the light period starting at 7 a.m. This project was approved by the Animal Experimentation Ethics Committee of the Faculty of Medicine of Jundiaí (protocol 62/2015).

**FIGURE 1:**
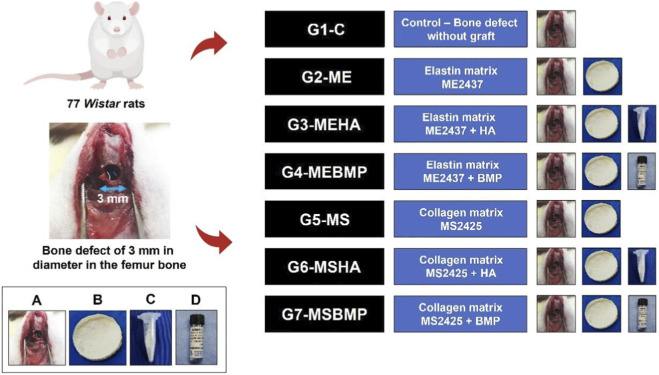
Schematic representation - Division of study groups and biomaterials used **(A)** Bone defect **(B)** Elastin or collagen membrane **(C)** Fine granules of hydroxyapatite **(D)** Bone morphogenetic protein.

The animals were divided into seven groups, with 11 animals each, and distributed as follows (Figure 1): Group 1 (G1-C) Control: Rats with a defect in the right femur and no filling; Group 2 (G2-ME): Rats with a defect in the right femur, filled with elastin membrane; Group 3 (G3-MEHA): Rats with a defect in the right femur, filled with elastin membrane + hydroxyapatite; Group 4 (G4-MEBMP): Rats with a defect in the right femur, filled with elastin membrane + BMP-2; Group 5 (G5-MS): Rats with a defect in the right femur, filled with collagen membrane from pig intestinal serosa; Group 6 (G6-MSHA): Rats with a defect in the right femur, filled with collagen membrane + hydroxyapatite; Group 7 (G7-MSBMP): Rats with a defect in the right femur, filled with collagen membrane + BMP-2.

From each group, six rats were allocated to histological analyses. Sample size was determined by *a priori* power analysis, considering a statistical power (β) of 0.90 and a significance level (α) of 0.05. Samples consisting of six specimens per group have demonstrated adequate statistical power in studies evaluating the osteoregenerative potential of experimentally created bone defects in rats (Samadian et al., 2021; Aroni et al., 2019). An additional five rats were allocated to biomechanical testing. For this analysis, the sample size was defined based on the statistical power confirmed in a previous study (Iatecola et al., 2021), which adopted a power (β) of 0.80 and a significance level (α) of 0.05.

### 2.1.6 Surgical procedures

**FIGURE 2:**
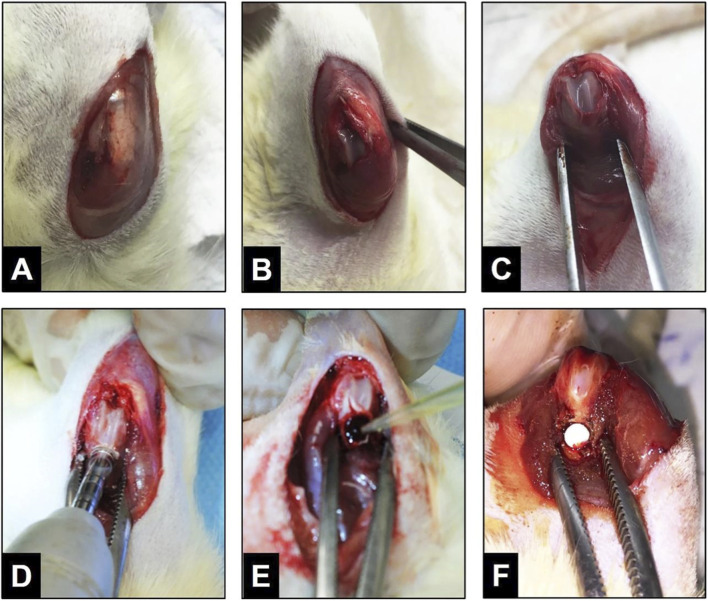
**(A)** Skin incision on the rat’s hind limb **(B)** Medial para-patellar incision **(C)** Exposure of the distal third of the femur **(D)** Creation of a 3 mm diameter bone defect with a trephine drill **(E)** Deposition of BMP-2 or hydroxyapatite granules **(F)** Grafting process with elastin or collagen membrane.

The animals were anesthetized with a combination of Xylazine and Ketamine. After preparing the surgical site with aseptic techniques, a midline incision was made on the right thigh to access the patella and extensor mechanism. A 3 mm bone defect was created in the anterior supracondylar region using a trephine drill, reaching the medullary canal. The area was irrigated with saline to prevent tissue damage. After creating the defect, the medullary content was removed, and the studied substances were implanted, except in the control group. Gauze soaked in saline was placed on both eyes to prevent corneal dehydration during surgery (Figure 2).

For groups G3-MEHA and G6-MSHA, following creation of the bone defect and thorough cleaning and drying of the medullary canal, 15 mg of hydroxyapatite (HA) was uniformly applied to the recipient site. This step was followed by placement of the bovine auricular cartilage–derived elastin matrix (ME) or the porcine intestinal serosa–derived collagen matrix (MS). Subsequently, an additional 15 mg of HA was applied over the matrix, resulting in a total of 30 mg of HA per graft. For groups G4-MEBMP and G7-MSBMP, recombinant human bone morphogenetic protein-2 (rhBMP-2) expressed in HEK-293 cells (HumanKine®, Sigma-Aldrich®) was used. The total vial content (10 µg) was dissolved in 2.5 mL of sterile saline solution. Using a micropipette, 275 µL of the resulting solution, corresponding to 1.1 µg of rhBMP-2 per animal, was collected. The grafting procedure followed the same sequence described above. Initially, 137 µL of the rhBMP-2 solution was deposited into the medullary canal, followed by placement of the ME or MS matrix. The remaining 138 µL was then applied over the matrix. The selected rhBMP-2 dose was based on previous studies demonstrating effective osteogenic stimulation in rat femoral bone defects at doses equal to or greater than 1.1 µg (Liu et al., 2016).

#### 2.1.7 Suturing, medication, and postoperative care summary

After surgery, the tissues were sutured using 5.0 silk thread, and the animals were given a dose of 0.1 mg/100 g of the antibiotic Pentabiotic, along with Rifamicina spray for the surgical wound. They also received oral paracetamol (200 mg/kg) for pain relief. Postoperatively, the animals were isolated in cages with regular food and water, and their bedding was changed several times a week. They were monitored for stress and pain management. At 6 weeks post-surgery, the animals were euthanized with a high dose of anesthetic and pneumothorax. The surgical areas were then analyzed for bone repair.

#### 2.1.8 Histological analysis

The histological analysis of the samples involved several steps to assess bone regeneration after implant use. The samples were fixed in a bone marrow fixative for 10 days and then decalcified for 7–10 days, with neutralization of the acid’s action. Next, macroscopic reduction was performed to isolate the grafted area, and the bone block was processed through a series of steps: dehydration in alcohols of varying concentrations, clarification with xylene, and embedding in paraffin.

After obtaining 5 μm sections, the samples were deparaffinized and stained with Hematoxylin and Eosin (H&E), Masson’s Trichrome, and Picrosirius Red. The staining allowed the distinction between original and newly formed bone, analysis of collagen fibers, and assessment of bone regeneration. Polarized light was used to observe different types of collagens (I, II, and III), providing detailed information on the extracellular matrix and osteoblastic differentiation stages.

#### 2.1.9 Radiological analysis

The samples were radiographed using an Odel equipment, digital processor CR-30X® AGFA (Agfa Healthcare–United States) with a technique of 300 mA, 0.05 mA s, and 40 kV for all samples. The objective was to visually assess the integrity of the bone defect (lesion), as well as the regeneration through the radiopaque and radiolucent appearance of the implant area.

#### 2.1.10 Morphometric analysis

Morphometric analysis was performed on histological sections stained with Masson’s Trichrome. Images were acquired under transmitted light microscopy using a standardized magnification. Digital image analysis was conducted using Motic Images Plus 2.0 software (Motic Digital Microscopy™, Kowloon, Hong Kong). The total area of the bone defect was first delineated based on the cortical margins of the recipient site. Subsequently, the area occupied by newly formed bone within the defect region was quantified using the software’s selection tools.

For each specimen, morphometric measurements were obtained from three distinct regions within the experimental area to account for regional variability. The percentage of newly formed bone was calculated as the ratio between the area of newly formed bone and the total defect area. Mean values were determined for each animal and subsequently for each experimental group.

All morphometric analyses were performed by a blinded examiner following a standardized protocol to minimize observer bias and ensure reproducibility.

#### 2.1.11 Biomechanical analysis

The maximum load capacity of the obtained anatomical pieces was studied using a three-point bending test. The femurs were dissected and kept refrigerated in saline solution.

The samples were thawed until reaching room temperature (19 °C–21 °C) and positioned in an INSTRON universal testing machine, model 4,444, with the proximal and distal ends of the femur supported on two rollers with a diameter of 3 mm, spaced 21.70 mm apart. The machine was programmed with a maximum load cell of 100 kgf (1 kN). The test was initiated with a pre-load of 5 N using a cylindrical rod with a 3 mm roller at its end, in the postero-anterior direction and perpendicular to the longitudinal axis, for sample accommodation and femur stabilization. The test allowed for a 15-s accommodation period of the samples in the equipment before applying a unidirectional force at a constant speed of 0.5 cm/min until the moment of bone fracture.

The data regarding the Maximum Load (kN) were recorded by the Instron Series IX software in graphical form. The kN corresponds to the highest load supported by the sample during the test.

### 2.1.12 Data analysis

For morphometric data analysis, histological sections stained with Masson’s Trichrome were examined under transmitted light microscopy. Initially, the total area of the noncritical bone defect was calculated based on the margins and cortical thickness of the recipient bed. After determining the total area, newly formed bone within the experimental area was quantified using the selection tool of the Motic Images Plus 2.0 software (Motic Digital Microscopy™, Kowloon, Hong Kong). The ratio between the total volume of newly formed bone and the total area of the noncritical bone defect was used to determine the percentage of new bone formation. Quantification for each specimen was performed through morphometric analysis of three distinct regions along the experimental area, yielding a mean percentage of newly formed bone for each rat and, subsequently, for each experimental group. For biomechanical testing, data related to maximum load (kN) were considered. Mean values and standard deviations were calculated, followed by statistical analysis of the results. Morphometric and biomechanical data were statistically analyzed using BioEstat 5.3™ software. Data were subjected to inferential analysis, initially by distribution testing and subsequently by one-way analysis of variance (ANOVA), followed by Tukey’s *post hoc* test, adopting a significance level of p < 0.05.

## 4 Results

### 4.1 Characterization

The integrity of the triple helix structure of the tropocollagen molecule was analyzed using DSC (Differential Scanning Calorimetry), by determining the denaturation temperature (Td), which corresponds to the collagen→gelatin transition. In DSC analysis, this transition appears as a discontinuity in the baseline, which is proportional to the difference in heat flow between the sample and the reference before and after denaturation. Table 1 presents the Td values for matrices derived from serosa and auricular cartilage, before (MSNAT and MENAT, respectively) and after alkaline treatment (MS24 and ME24, respectively). The presence of the transition indicates the denaturation of residual collagen within the elastin matrix, even after alkaline treatment at 37 °C.

**TABLE 1:** Denaturation temperature (Td, °C) for the different matrices.

Matrix	Td (°C)
MENAT^a^	75.2
ME	57.2
MSNAT^b^	70.1
MS	60.8

^a^ Auricular cartilage without alkaline treatment.

^b^ Serosa membrane without alkaline treatment.

The matrices showed a decrease in Td after alkaline hydrolysis when compared to the native biological tissues. This is due to the breakdown of cross-linking bonds and changes in the charge distribution within the tropocollagen, which lead to fiber disorganization and reduced structural stability.

**FIGURE 3:**
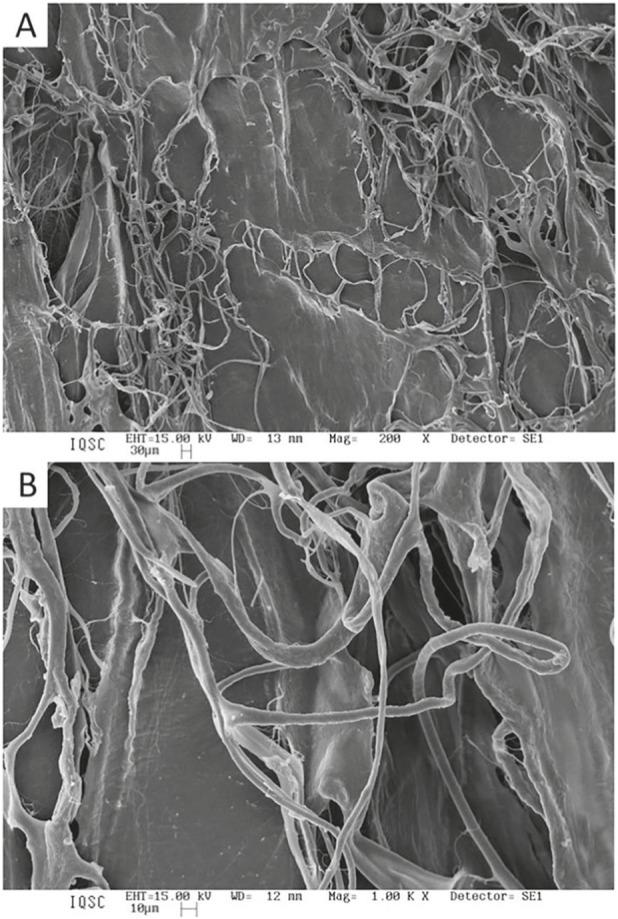
SEM micrograph of elastin matrix after alkaline treatment. Magnification of **(A)** ×200; **(B)** ×1,000.

**FIGURE 4:**
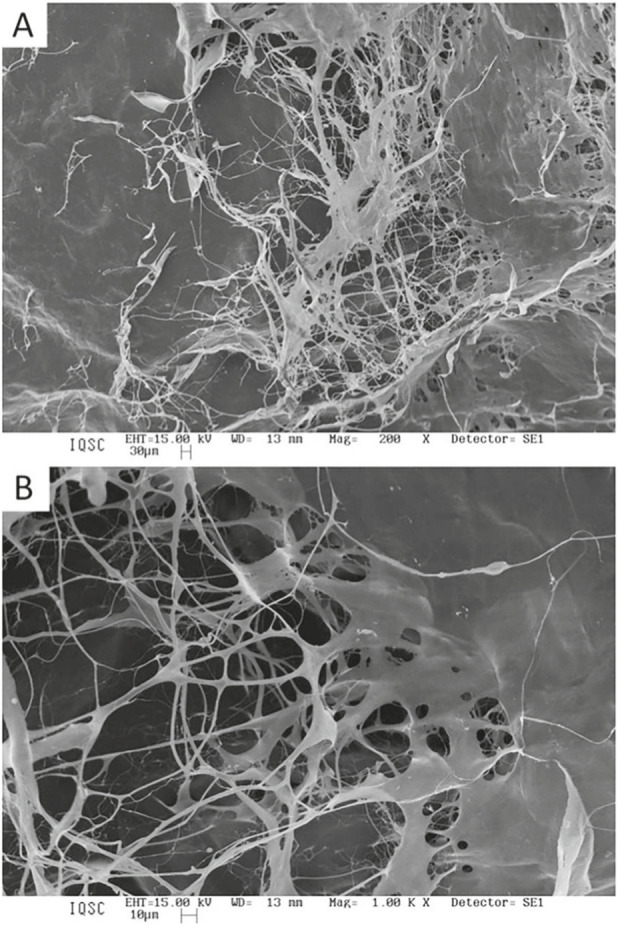
SEM micrograph of porcine serosa matrix after alkaline treatment. Magnification of **(A)** ×200; **(B)** ×1,000.

The matrices were also characterized by scanning electron microscopy (SEM), which revealed rough surfaces with visible fibers for both types of matrices (Figures 3, 4). Although the original materials are different, the alkaline treatment at specific temperatures resulted in morphologically similar matrices.

#### 4.2 Morphometric results

**FIGURE 5:**
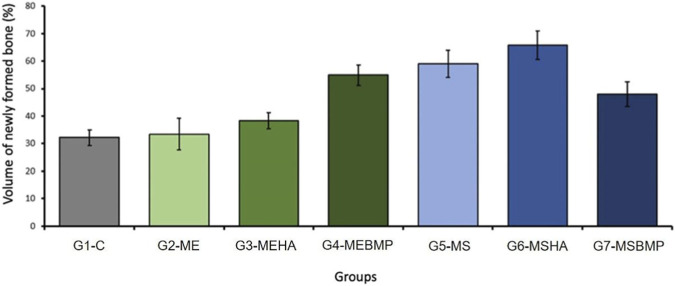
Graph of the morphometric analysis of groups G1 to G7. Demonstration of the percentage of newly formed bone in the surgical area.

The means and standard deviations of the relative percentage volume of newly formed bone in the experimentally created bone defect in the right femur of rats, sacrificed 6 (six) weeks post-operation (G1 to G7), were as follows: 32.221 ± 2.809; 33.442 ± 5.743; 38.328 ± 2.845; 54.859 ± 3.723; 59.0148 ± 5.0328; 65.726 ± 5.197; 47.911 ± 4.507, as shown in Figure 5.

There was a significant difference with a significance index of p < 0.01 for groups G1, G2, and G3, compared to groups G4, G5, G6, and G7. A statistically greater difference (p < 0.05) was identified between groups G3 and G7.

**FIGURE 6:**
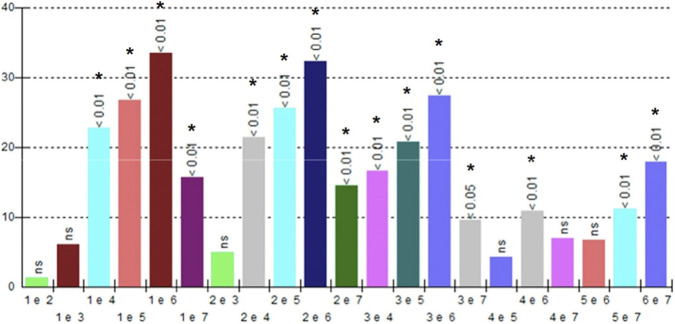
Graph of the statistical analysis of groups G1 to G7. Statistical differences among groups are shown. Statistical significance is indicated using standard symbols: *p < 0.05, **p < 0.01; ns indicates no statistically significant difference.

Although the experimental groups G2 and G3 showed a higher percentage of new bone formation compared to the control group G1, the statistical analysis did not identify a significant difference (ns), (Figure 6).

#### 4.3 Biomechanical test results

**FIGURE 7:**
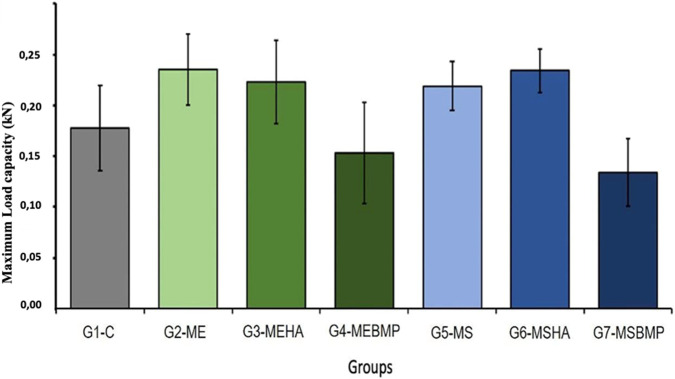
Graph of the biomechanical analysis of groups G1 to G7. Demonstration of the maximum load capacity supported.

The means and standard deviations of the Maximum Load capacity (kN) supported during the biomechanical test of groups G1 to G7 were as follows: 0.178 ± 0.042; 0.235 ± 0.035; 0.223 ± 0.041; 0.153 ± 0.050; 0.219 ± 0.024; 0.234 ± 0.021; 0.134 ± 0.033, as shown in Figure 7.

**FIGURE 8:**
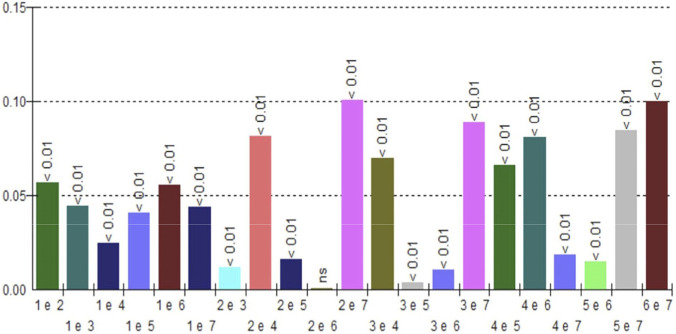
Graph of the statistical analysis of the maximum load of groups G1 to G7.

The maximum load data were subjected to intergroup statistical analysis, which identified significant statistical differences (p < 0.01). Only the data between groups G2 and G6 did not show a difference with a relevant significance index (ns), (Figure 8).

#### 4.4 Radiological analysis of the surgical area

**FIGURE 9:**
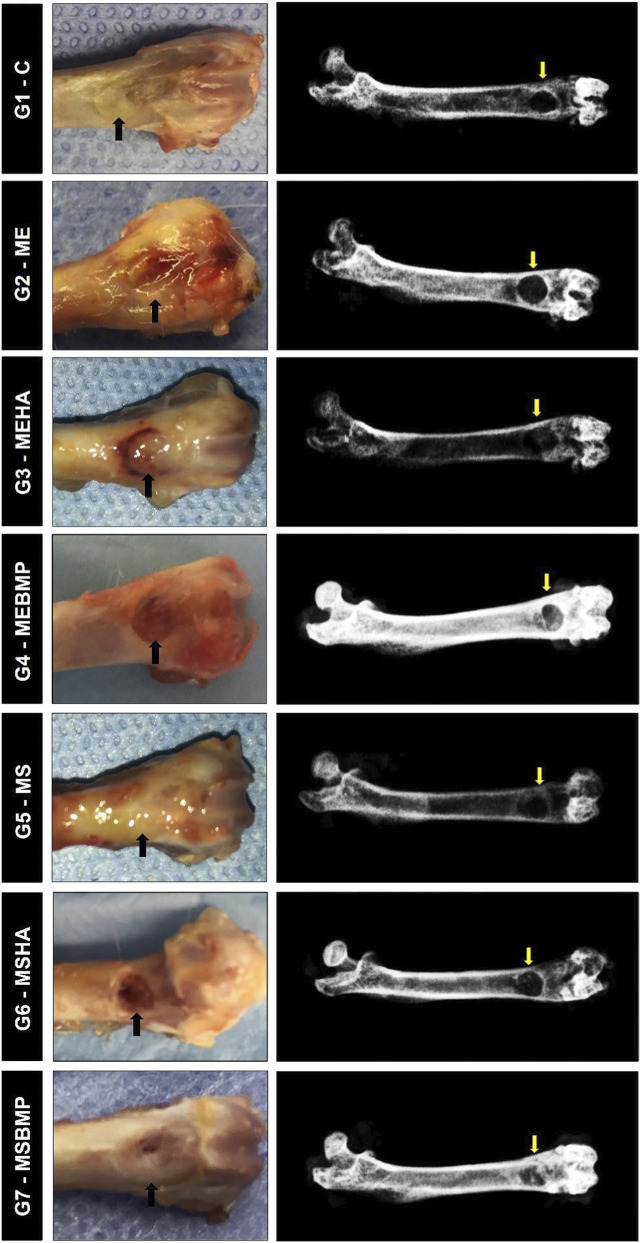
Photomacrography and radiology of the study groups. On the left, macroscopic images of the surgical areas of groups G1 to G7. On the right, radiographs of groups G1 to G7.

In all the groups studied, no radiological signs of pathological alterations were observed in the grafted area, suggesting the compatibility of the biomaterial with the adjacent bone tissue. It was also noted that the normal architecture of the femur was maintained, with preservation of the cortical bone at its medial and lateral margins. There were no deformities or other complications such as non-union or secondary fractures due to weight-bearing and animal locomotion. It was also observed that the radiopaque definition of the edges of the experimentally created bone defect was present, with radiolucency in the interior of the defect, indicating that there was proliferation of connective tissue and not complete repair through the necessary amount of newly formed bone during the bone regeneration process. In the central region of the bone defect, the characteristic radiotransparency still remained, but new bone was projected from its borders, marked by the radiopaque image (Figure 9).

In the groups grafted with the biomaterials, it was not possible to identify a more radiopaque image of the biomaterial that could indicate associated new bone formation, due to the low atomic weight of the immature bone, making it imperceptible in the radiological image.

#### 4.5 Histological analysis of the femoral surgical area

Histological findings from all groups with biomaterial showed the formation of subperiosteal bone tissue, extending from the original bone towards the bone defect, creating a bony bridge. Newly formed bone varied across groups in terms of quantity and maturity. Trabeculae migrated to the center of the medullary canal, surrounded by connective tissue, with irregular geometry. Central immature areas stained blue, showing disorganized osteocyte lacunae, while peripheral lamellar areas stained red, with aligned lacunae, indicating remodeling. The boundary between existing and newly deposited bone was clear, marked by the basophilic reversal line. No signs of fibrous formations or inflammatory infiltration were found, ruling out immune rejection. Granulation tissue with immature mesenchymal cells was observed in the biomaterial groups, particularly in areas without new bone formation, where connective tissue was present in the superficial regions. Picrosirius Red (PSR) staining revealed a red-to-yellow gradient in the bone defect area, indicating type I collagen fibers, while type III collagen fibers, indicated by green staining, were rarely seen in the implant groups.

**FIGURE 10:**
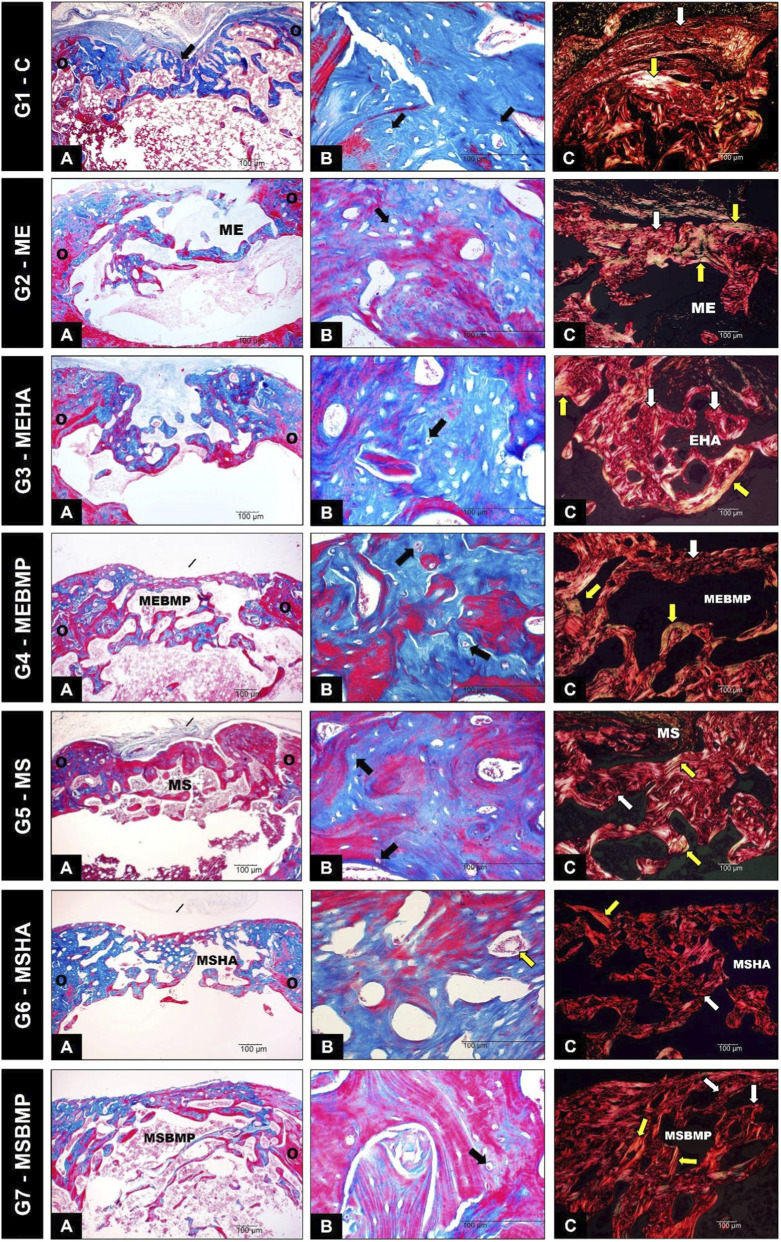
Photomicrographs of Groups G1 to G7 **(A)** Masson’s Trichrome Staining at ×40 magnification **(B)** Masson’s Trichrome Staining at ×400 magnification (O) Indicates the edges of the receptor area (Black arrow) Indicates the presence of osteocytes lodged in lacunae (Yellow arrows) Indicate the presence of HA granules **(C)** Picrosirius Red Staining at ×100 magnification (White arrows) Indicate the presence of disorganized collagen fibers in red tones (Yellow arrows) Indicate the presence of dense and parallel collagen fibers in the tissue organization phase, with colors ranging from yellow to greenish (ME) Indicates the presence of elastin membrane (MS) Indicates the presence of collagen membrane.

Group 1 (G1-C) Control: The formation of a bony bridge uniting the two ends of the bone defect was not evident. There was a predominance of newly formed bone with an immature appearance, consisting of thin trabeculae. The newly formed bone showed birefringence of its collagen fibers in orange and red tones, indicating type I collagen in the regions near the edges of the lesion, predominantly, and thin, dispersed connective tissue superficially in greenish tones, indicating type III collagen.
Group 2 (G2-ME): In this group, the migration of newly formed bone was observed from the basophilic reversal line, both from the superficial (periosteal) region and the central (endosteal) region. However, a faster degradation rate compared to new bone formation was noted, represented by the abundant gaps between the newly formed bone. Immature bone tissue predominated in this group, in the form of irregular trabeculae. The non-absorbed membrane between the newly formed trabeculae and in the medullary canal was observed. The newly formed bone predominantly showed birefringence of its collagen fibers in orange and red tones, indicating type I collagen. Discrete greenish fibers were also present in the periosteal region, characteristics that would represent type III collagen.
Group 3 (G3-MEHA): In this group, there was greater formation of immature tissue with thin and irregular trabeculae, predominantly endosteal with a lower periosteal concentration. A tendency for the formation of a bony bridge through the central region of the defect was observed. Rapid degradation of the biomaterial occurred, but with increased formation of new bone, with small gaps in the formation process. Small quantities of HA particles were observed in the composition of the newly formed tissue. On the surface of the defect, abundant irregular connective tissue was evident. The newly formed bone showed birefringence of its collagen fibers in orange and red tones, indicating type I collagen predominantly in the central region of the defect, and the presence of green-toned fibers in the superficial region, representing type III collagen.
Group 4 (G4-MEBMP): In this group, the complete formation of a bony bridge between the edges of the defect was observed. There was also abundant formation of newly formed bone tissue, both on the surface (periosteal) and endosteal, with characteristics of mature bone, including the formation of regular trabeculae in the medullary canal. A greater balance between the absorption of the biomaterial and the formation of new bone occurred, although irregular gaps remained between the new bone formation and the remnants of the membrane. The newly formed bone showed birefringence of its collagen fibers in red and orange tones, indicating type I collagen predominantly in the superficial region of the defect. Dispersed greenish foci were observed in the central region, representing type III collagen.
Group 5 (G5-MS): A prominent bony bridge uniting the edges of the defect was evident. There was a predominance of newly formed bone tissue with a mature appearance, with the presence of the membrane scattered throughout the new bone tissue. The newly formed bone showed birefringence of its collagen fibers in red and orange tones, indicating type I collagen predominantly in the central region of the defect. Dispersed greenish foci were observed in the superficial region, representing type III collagen.
Group 6 (G6-MSHA): The formation of a bony bridge between the edges of the defect was observed, with a predominance of newly formed bone tissue with a mature appearance and immature tissue in smaller amounts. Within the newly formed bone, the presence of hydroxyapatite granules was noted, surrounded and in direct contact with the formed bone. Remnants of the membrane were observed in the central region of the bone defect, with connective tissue on the surface. The newly formed bone showed intense birefringence of its collagen fibers in red and orange tones, indicating type I collagen in the largest region of the new bone formation. Small and dispersed greenish foci were observed in the central region, representing type III collagen.
Group 7 (G7-MSBMP): In this group, there was a predominance of newly formed bone tissue with a mixed appearance (mature/immature) at the edges of the bony bridge. In the central part of the medullary canal, mature bone trabeculae were present. An excellent balance between bone absorption and the formation of new tissue occurred, characterized by consistent new bone formation with no gaps. The newly formed bone showed birefringence of its collagen fibers in red and orange tones, indicating type I collagen predominantly in the superficial region of the defect. The rare presence of small and dispersed greenish foci in the central region was observed, representing type III collagen (Figure 10).

## 5 Discussion

The repair of critical-sized bone defects remains one of the major challenges in orthopedic regenerative medicine due to the limited self-healing capacity of bone when the defect exceeds a critical threshold. In this context, the development of biomaterials that support cell migration, osteogenic differentiation, and bone matrix deposition is crucial (Wang et al., 2024). This study evaluated the effects of collagen and elastin matrices—alone and in combination with hydroxyapatite (HA) or bone morphogenetic protein-2 (BMP-2)—on bone regeneration in rat femoral defects. The results demonstrated that the collagen membrane derived from porcine serosa, especially when combined with BMP-2, significantly enhanced bone formation, suggesting its superior suitability for scaffold-based bone repair strategies (Zhang et al., 2020). The most relevant histological and biomechanical outcomes were observed in groups treated with collagen + HA and collagen + BMP-2 (Dec et al., 2022).

A key property that directly influences the success of biomaterials in bone repair is porosity, which impacts not only the degree of cellular migration and tissue integration but also the scaffold’s vascularization and degradation profile. It is well established that the ideal pore size for scaffolds intended for bone regeneration lies between 100 and 500 μm (de Moraes et al., 2023; Cunha et al., 2021). Pores within this range permit cell infiltration, neovascularization, nutrient diffusion, and effective bone tissue ingrowth while preserving sufficient mechanical strength to support the healing process. In our SEM analysis, both elastin and collagen matrices exhibited micro- and macroporous surfaces; however, the collagen matrix showed more consistent interconnectivity and surface roughness, factors that are directly linked to enhanced osteoconduction and osteointegration (Munhoz et al., 2020; Wei et al., 2018; Karageorgiou and e Kaplan, 2005). The elastin matrix, while also porous, appeared denser and less interconnected, which may have limited cellular colonization and the subsequent osteogenic process. DSC analysis demonstrated a reduction in denaturation temperature (Td) after alkaline treatment, indicating partial disruption of collagen cross-links and decreased structural stability. Although Td values do not directly predict *in vivo* performance, they provide relevant information on matrix modification and potential susceptibility to biodegradation under physiological conditions (Zhang et al., 2022; Picado-Tejero et al., 2025).

When analyzing each biomaterial separately, the elastin matrix, derived from bovine auricular cartilage and treated under mild alkaline conditions, demonstrated good biocompatibility (Mukasheva et al., 2024). No inflammatory infiltrates were observed in the histological analysis, and the material remained partially intact 6 weeks post-implantation. However, the groups receiving elastin alone (G2-ME) or with HA (G3-MEHA) exhibited limited bone bridging, with predominance of immature bone and the presence of residual gaps within the defect (Gupte et al., 2018; Koushik et al., 2023; Sawadkar et al., 2021). This indicates that although elastin supports initial cell adhesion and offers structural support, its faster degradation, lower porosity, and possibly less favorable surface chemistry may impair sustained osteogenesis (Amruthwar and e Janorkar, 2013; Flaig et al., 2020).

Interestingly, when elastin was combined with BMP-2 (G4-MEBMP), a marked increase in bone formation was observed, including the formation of more regular trabeculae and improved periosteal and endosteal bone bridging (Johnson et al., 2011). This is consistent with previous findings suggesting that elastin can act as a carrier for BMP-2, allowing a biphasic release pattern—an initial burst followed by a delayed release due to the hydrophobic nature of elastin at body temperature. Furthermore, elastin degradation products may act as signaling molecules, promoting the release of TGF-β1 and the upregulation of genes related to osteogenesis and angiogenesis. These molecular mechanisms may explain the improved bone quality observed in G4 (Lauer et al., 2020; Gao et al., 2023; Iatecola et al., 2021).

In contrast, the collagen membrane, processed from porcine intestinal serosa and treated under controlled alkaline hydrolysis at 25 °C, displayed superior biological performance. Histologically, the groups receiving collagen alone (G5-MS) or in combination with HA (G6-MSHA) and BMP-2 (G7-MSBMP) showed abundant new bone formation, well-organized trabeculae, and the presence of both immature and mature lamellar bone. The birefringence under polarized light, especially in G6 and G7, confirmed the deposition of type I collagen and progressive bone remodeling (de Oliveira et al., 2024; de Moraes et al., 2019; McCarthy et al., 2016). The G7 group, in particular, exhibited the most advanced stage of bone repair, with nearly complete defect closure and minimal gaps—highlighting the synergistic effect of collagen and BMP-2 (Joyce et al., 2021; Sarangthem et al., 2022).

Biomechanical data further supported these findings. The highest maximum load values were recorded in groups treated with collagen-based scaffolds, especially G6 (collagen + HA), indicating that the new bone not only filled the defect but also contributed to functional mechanical strength. This mechanical integrity is essential for load-bearing applications and long-term clinical success (Ramos et al., 2024; Baggio et al., 2024).

The combination of hydroxyapatite and collagen proved particularly effective. HA, a bioceramic with chemical and structural similarity to bone mineral, enhances osteoconduction and provides mechanical support (Astaneh et al., 2025; Pettian et al., 2018; Rocha et al., 2002; Kane et al., 2015). When integrated into the collagen scaffold, HA particles were observed to be in direct contact with the newly formed bone, suggesting active participation in the mineralization process. Additionally, HA has a high affinity for collagen and facilitates the adsorption of bone-related growth factors, which may have contributed to the robust osteogenesis seen in G6 (Patel et al., 2019; Kołodziejska et al., 2020; Wahl and e Czernuszka, 2006; Xie et al., 2023).

The histological findings consistently showed that the presence of a scaffold, especially when combined with BMP-2 or HA resulted in more organized bone formation and greater trabecular density (Patel et al., 2019; Kołodziejska et al., 2020). Picrosirius Red staining confirmed that most of the newly formed bone was composed of type I collagen, with only sparse type III collagen, suggesting a maturation of the extracellular matrix. The presence of a clear reversal line between native and new bone in all treated groups further supports the effective integration of the grafts (Cholas et al., 2016; Xu et al., 2023).

Although the BMP-2–treated groups presented higher volumes of newly formed bone, they exhibited lower maximum load values in the biomechanical analysis. This apparent discrepancy highlights that increased bone volume does not necessarily translate into superior mechanical performance. BMP-2 is known to promote rapid osteogenesis, often resulting in bone tissue with a higher proportion of woven, less organized structure and reduced mineral density, particularly at early or intermediate healing stages. Consequently, although morphometric outcomes indicate enhanced bone formation, the newly formed tissue may not yet possess the microstructural organization required to support higher mechanical loads. These findings suggest that differences in bone maturation and quality, rather than bone quantity alone, likely underlie the observed biomechanical behavior at the 6-week time point (Zara et al., 2011; Boerckel et al., 2011).

Despite these encouraging results, this study has some limitations. The observation period was limited to 6 weeks, which, although sufficient to assess bone formation and biomechanical outcomes in a non-critical defect model, may not fully capture the temporal progression of healing, biomaterial degradation, long-term remodeling, or the final mechanical properties of the regenerated bone (Geng et al., 2021; López De Padilla et al., 2021). In addition, quantitative porosity analysis and *in vivo* degradation profiles of the matrices were not evaluated and should be addressed in future studies to better correlate material properties with biological performance. Furthermore, histological evaluation was primarily qualitative, and the absence of semi-quantitative scoring systems or additional histomorphometric parameters may limit a more detailed assessment of inflammatory response, vascularization, and bone maturation. Another limitation of this study is the lack of direct comparison with commercially available bone substitute materials, which are commonly used as benchmarks in bone tissue engineering research. Finally, although no adverse reactions were observed, the use of animal-derived matrices may pose translational challenges related to potential immunogenicity and material variability. Future studies employing critical-sized defect models, commercially available reference materials, and multiple time points are warranted to further elucidate these dynamic processes (Liu et al., 2021; Pereira et al., 2020).

Clinically, the results suggest that collagen-based scaffolds, particularly when combined with BMP-2 or HA, are promising candidates for the treatment of critical bone defects. Their ability to support cellular migration, matrix deposition, and mechanical recovery makes them suitable for use in maxillofacial surgery, orthopedics, and reconstructive procedures (Kasravi et al., 2023). From a translational perspective, future research should explore modifications to optimize degradation rates, enhance bioactivity, and possibly integrate smart drug-delivery systems or 3D-printed architectures tailored to defect morphology (Rybachuk et al., 2024; Yang et al., 2011; Tang et al., 2025; Reis et al., 2026).

Therefore, the biomechanics of bone tissue plays a pivotal role in ensuring the durability and performance of orthopedic implants, particularly through the mechanical integrity of medium-viscosity PMMA bone cements under physiological stresses. Karpiński et al. (Karpiński et al., 2022) showed that intraoperative contaminants like blood and saline significantly affect compressive strength and elastic modulus, with low concentrations (up to 2%) enhancing properties while higher levels (over 8%) lead to sharp declines, emphasizing the need for meticulous surgical handling. Complementing this, Szabelski et al. (Szabelski et al., 2024a) found that incorporating α/β-tricalcium phosphate (TCP) above 3% diminishes compressive strength—especially with α-TCP—though β-TCP preserves baseline mechanics while supporting osteogenesis. Similarly, Szabelski et al. (Szabelski et al., 2024b) reported that 2% hydroxyapatite addition subtly modifies PMMA’s mechanical profile, boosting bioactivity without major compromises to load-bearing capacity in implant scenarios.

In conclusion, collagen and elastin matrices demonstrated favorable biocompatibility and supported the bone regeneration process in critical-sized femoral defects in rats. However, the collagen matrix, especially when combined with BMP-2, led to the most significant improvements in both bone volume and mechanical resistance, likely due to its optimized porosity, osteoconductive properties, and capacity for sustained release of osteoinductive signals. These findings pave the way for further refinement and clinical translation of biologically inspired scaffolds for bone tissue engineering.

## Data Availability

The original contributions presented in the study are included in the article/supplementary material, further inquiries can be directed to the corresponding author.
